# Supplementary material for: Excess mortality by specific causes of deaths in the city of São Paulo, Brazil, during the COVID-19 pandemic
Source: PLoS One. 2021 Jun 7;16(6):e0252238. doi: 10.1371/journal.pone.0252238 (PMC8184000; doi:10.1371/journal.pone.0252238)
Supplement: S1 Table — (DOCX) [file pone.0252238.s001.docx]

S1 Table - Causes of deaths and corresponding ICD-10 codes

| **Causes of Death** | **X** |
| --- | --- |
| All Causes | A00-T98 |
| Diabetes mellitus | E14 |
| Cardiovascular diseases | I00-I99 |
| Cerebral vascular accident | I64 |
| Acute myocardial infarction | I21 |
| Congestive heart failure | I50 |
| COVID-19 | B34.2 |
| All cancers | C00-97 |
| Lung | C33-34 |
| Breast | C50 |
| Colorectum | C18-21 |
| Prostate | C61 |
| Stomach | C16 |
| Liver | C22 |
| Oesophagus | C15 |
| Cervix uteri | C53 |
| Thyroid | C73 |
| Bladder | C67 |
| Non-Hodgkin lymphoma | C82-86, C96 |
| Pancreas | C25 |
| Leukaemia | C91-95 |
| Kidney | C64-65 |
| Corpus uteri | C54 |
| Lip, oral cavity | C00-06 |
| Brain, central nervous system | C70-72 |
| Ovary | C56 |
| Melanoma of skin | C43 |
| Gallbladder | C23-24 |
| Larynx | C32 |
| Multiple myeloma | C88+C90 |
| Nasopharynx | C11 |
| Oropharynx | C09-10 |
| Hypopharynx | C12-13 |
| Hodgkin lymphoma | C81 |
| Testis | C62 |
| Salivary glands | C07-08 |
| Vulva | C51 |
| Penis | C60 |
